# Tumor Ulceration, Reduced Infiltration of CD8-Lymphocytes, High Neutrophil-to-CD8-Lymphocyte Ratio and Absence of MC Virus are Negative Prognostic Markers for Patients with Merkel Cell Carcinoma

**DOI:** 10.3390/cancers12040888

**Published:** 2020-04-06

**Authors:** Simon Naseri, Torben Steiniche, Jeanette Bæhr Georgsen, Rune Thomsen, Morten Ladekarl, Martin Heje, Tine Engberg Damsgaard, Marie Louise Bønnelykke-Behrndtz

**Affiliations:** 1 Department of Plastic Surgery, Aalborg University Hospital, 9000 Aalborg, Denmark; 2 Department of Pathology, Aarhus University Hospital, 8200 Aarhus, Denmark; torbstei@rm.dk (T.S.); jeanette.georgsen@aarhus.rm.dk (J.B.G.); 3 Department of Biomedicine, Aarhus University, 8200 Aarhus, Denmark; rt@biomed.au.dk; 4 Department of Oncology, Clinical Cancer Research Center, Aalborg University Hospital, 9000 Aalborg, Denmark; morten.ladekarl@rn.dk; 5 Department of Plastic Surgery, Vejle Hospital, 7100 Vejle, Denmark; martin.heje@dadlnet.dk; 6 Department of Plastic Surgery and Burns Treatment, Rigshospitalet, 2100 Copenhagen, Denmark; tinemed@gmail.com; 7 Department of Plastic and Reconstructive Surgery, Aarhus University Hospital, 8200 Aarhus, Denmark; louiseboennelykke@gmail.com

**Keywords:** Merkel cell carcinoma, Merkel cell polyoma virus, tumor microenvironment, CD8 lymphocytes, ulceration, E-cadherin

## Abstract

(1) Background: Merkel cell carcinoma (MCC) is caused by the Merkel cell polyomavirus and UV radiation. Understanding of the underlying biology is limited, but identification of prognostic markers may lead to better prognostic stratification for the patients. (2) Methods: Ninety patients diagnosed with MCC (1996–2012) were included. Virus status was estimated by polymerase chain reaction (qPCR) and immunohistochemistry (IHC). Ulceration status, PD-L1, cd66b neutrophils, cd8 lymphocytes and biomarkers of vascularization (cd34 endothelial cells) and migration (e-cadherin) were estimated by IHC and analyzed with digital pathology. (3) Results: Virus was present in 47% of patient samples and correlated with lower E-cadherin expression (*p* = 0.0005), lower neutrophil-to-CD8 lymphocyte ratio (N:CD8 ratio) (*p* = 0.02) and increased PD-L1 expression (*p* = 0.03). Ulceration was associated with absence of virus (*p* = 0.03), increased neutrophil infiltration (*p* < 0.0001) and reduced CD8 lymphocyte infiltration (*p* = 0.04). In multivariate analysis, presence of virus (*p* = 0.01), ulceration (*p* = 0.05) and increased CD8 lymphocyte infiltration (*p* = 0.001) showed independent prognostic impacts on MCC-specific survival. (3) Conclusions: In this study, we found that a high N:CD8 ratio, ulceration, virus-negative status and absence of CD8 lymphocytes are negative prognostic markers. Accurate prognostic stratification of the patients may be important in the clinical setting for determination of adjuvant treatment.

## 1. Introduction

Merkel cell carcinoma (MCC) is a highly aggressive malignancy of the skin with a five-year overall survival rate of 40% [1]. MCC was first described by Toker in 1972 and has during the past decades shown an up to five-fold increase in incidences in western countries [2,3,4,5]. Although the cell of origin of MCC is still debated, the etiology is believed to be UV-radiation (20%) and the recently discovered Merkel cell polyomavirus (MCV) (80%) [6,7]. Despite its poor prognosis, recent clinical trials with immune therapy with checkpoint inhibitors show high response rates, exceeding response rates observed in most other solid tumors. The reason for this might be rooted in the inflammatory microenvironment [8,9,10]. In most solid tumors, the tumor microenvironment (TME) plays an essential role in both tumor growth and dissemination but also in response to treatment [11]. However, a characterization and understanding of the TME is limited and still largely undescribed in MCC.

Both viral status (MCV-positive or negative) [12] and infiltrating immune cells (e.g., neutrophils and CD8 lymphocytes) [13,14,15] can be pivotal contributors to either a pro- or anti-TME, which in turn may impact the migratory functions of the tumor cells (e.g., assessed by loss of E-cadherin) [16,17,18] and response to immune checkpoint inhibitors (generally enhanced in tumors with PD-L1 expression) [19]. In addition, one of the leading prognostic factors in other skin malignancies like melanoma is ulceration [20], which we have previously shown is linked to a tumor-supportive microenvironment [16,17]. We aim to study in MCC the interaction between tumor cell viral status, ulceration and the microenvironment (assessed by PD-L1, E-cadherin, endothelial cells and immune cell stain densities), aiming for a better understanding of these factors that may play an essential role in both the natural and treatment-related biology of MCC.

## 2. Results

### 2.1. Ulceration in MCC Is Associated with Increased Infiltration of Neutrophils and Decreased Infiltration of CD8 Lymphocytes

Ulceration was present in 29.5 % (*n* = 23) of primary tumors and absent in 70.5 % (*n* = 55). The remaining tumors could not be evaluated due to missing epidermal regions in the tumor sections (*n* = 12). There was no difference in clinical characteristics between ulcerated and nonulcerated MCC (Table S1). Ulcerated tumors were characterized by increased (*p* < 0.0001) stain area fractions of neutrophils (0.02%; 95% CI: 0.00–0.90 vs. 0.06 × 10^−3^%; 95% CI: 0.02 × 10^−3^–0.18 × 10^−3^, Figure S1B,E) and an increased (*p* < 0.0001) neutrophil-to-CD8 lymphocyte ratio (N:CD8) (0.91; 95% CI: 0.12–6.92 vs. 0.33 × 10^−3^; 95% CI: 0.09 × 10^−3^–1.23 × 10^−3^ ), compared with nonulcerated tumors. In contrast, ulcerated tumors had lower (*p* = 0.04) stain area fractions of CD8 lymphocytes (0.02%; 95% CI: 0.00–0.10 vs. 0.19%; 95% CI: 0.06–0.60), compared with nonulcerated tumors (Figure S1C,F).

### 2.2. Ulceration Is Associated with Virus-Negative MCC

Virus was present in 47% (43/90) of the included MCC patient samples, while 53% (57/90) were virus-negative. Ulceration associated significantly with virus-negative MCC (*p* = 0.03) and was present in 39.5% (17/43) of the virus-negative MCC and only in 17.1% (6/35) of the virus-positive MCC. Ulceration did not associate with tumor size (*p* = 0.56).

### 2.3. Virus-Positive MCC Presents Higher Densities of PD-L1, Lower Neutrophil-to-CD8 Lymphocyte Ratio and Lower Density of E-Cadherin

Virus status was estimated with both qPCR and immunohistochemistry (IHC). Estimated by qPCR, 47% (43/90) of patients were virus-positive. Two additional patients had a positive PCR but were categorized as PCR-negative, as their viral primer/TBP ratio was below the 0.01 cut-off. Estimated by IHC, 40% (36/90) of patients were virus-positive. One additional patient had positive immune staining but was categorized as IHC-negative, as the stained cells were stromal cells. There was a high concordance between IHC and qPCR for virus detection (*p* < 0.0001), with IHC detecting 83.7% of qPCR-positive samples.

Patients with virus-positive MCC were younger (74.7 years vs. 80.8 years; *p* = 0.008), and the primary location of MCC varied significantly between the virus-negative and virus-positive groups (*p* = 0.006). Virus-positive primary tumors were primarily located on the extremities (60.5% vs. 27.6%), and the virus-negative tumors were more often located in the head-and-neck area (61.7% vs. 30.2%), while location on the trunk was rare but equally distributed between the groups (9.3% vs. 10.6%). Factors of the local TME in virus-positive and -negative MCC are illustrated in Table 1. Virus-negative MCC was significantly associated (*p* = 0.02) with an increased N:CD8 ratio (15.93 × 10^−3^; 95 % CI: 2.20 × 10^−3^–115.16 × 10^−3^), compared with virus-positive MCC (0.81 × 10^−3^; 95% CI: 0.16 × 10^−3^–4.12 × 10^−3^). Virus-positive MCC was significantly associated (*p* = 0.0005) with reduced stain area fractions of E-cadherin (0.27 × 10^−3^ %; 95% CI: 0.04 × 10^−3^–2.04 × 10^−3^), compared with virus-negative MCC (56.57 × 10^−3^; 95 % CI: 6.44 × 10^−3^–497.02 × 10^−3^, Figure S2D,H). In addition, presence of the virus associated (*p* = 0.03) with an increased stain area fraction of PD-L1 (59.28 × 10^−3^%; 95 % CI: 9.46 × 10^−3^–371.29 × 10^−3^), compared with virus-negative samples (4.36 × 10^−3^ %; 95 % CI: 0.84 × 10^−3^–22.68 × 10^−3^) (Figure S2C,G).

### 2.4. Density of CD8 Lymphocytes and PD-L1 Are Associated

Increasing stain area fractions of CD8 lymphocytes in the tumor (*p* < 0.0001) and a low N:CD8 ratio (*p* = 0.0003) associated with an increased PD-L1 stain area fraction.

### 2.5. Density of CD8 Lymphocytes, Neutrophil-to-CD8 Lymphocyte Ratio, Virus-Positive Status, Ulceration and Nodal Involvement Have Independent Impact on MCC Specific Survival

In univariate analysis, a significantly reduced MCC-specific survival was seen in patients with an ulcerated primary tumor (HR = 2.49; 95% CI= 1.18–5.25; *p* = 0.02), increased N:CD8 ratio (HR = 1.21; 95% CI= 1.06–1.37; *p* = 0.004) and nodal involvement (HR = 3.17; 95% CI = 1.47–6.81; *p* = 0.003). A significantly improved MCC-specific survival was seen in patients with an increased stain area fraction of CD8 lymphocytes (HR = 0.70; 95% CI= 0.57–0.87; *p* = 0.001) and with a positive viral status (HR = 0.47; 95% CI = 0.22–1.00; *p* = 0.05). No significant difference in MCC-specific survival was seen based on the stain area fraction of PD-L1 expression (*p* = 0.21), E-cadherin (*p* = 0.73), endothelia (*p* = 0.74), neutrophils (*p* = 0.32) or tumor size (*p* = 0.35). The results of the univariate analysis are illustrated in Table 2.

For the multivariate analysis, we chose to adjust for T-size over and under 2 cm and lymph node involvement, as these factors are known and accepted prognostic markers of MCC. Presence of ulceration (HR = 2.22; 95% CI= 0.99–4.98; *p* = 0.05) and an increased N:CD8 ratio (HR = 1.14; 95% CI = 1.00–1.31; *p* = 0.04) had negative independent prognostic impacts on MCC-specific survival. Kaplan-Meier survival curves for ulcerated and nonulcerated MCC are illustrated in Figure S1G. A significantly improved MCC-specific survival was seen in patients with an increased stain area fraction of CD8 lymphocytes (HR = 0.68; 95% conf. 0.54–0.85; *p* = 0.001) and with a positive viral status (HR = 0.32; 95% CI = 0.13–0.78; *p* = 0.01). Kaplan-Meier survival curves for virus-positive and -negative MCC are illustrated in Figure S2I. No significant difference in MCC-specific survival was seen based on the stain area fractions of PD-L1 (*p* = 0.29), neutrophils (*p* = 0.87), endothelia (0.77) or E-cadherin (*p* = 0.73). The results of the multivariate analysis are illustrated in Table 3.

## 3. Discussion

The primary aim of this study was to investigate prognostic markers of MCC, an aggressive skin tumor with worse prognosis than melanoma [21]. We collected the majority of primary MCC samples from patients diagnosed between 2007–2012 in Denmark. We aimed to characterize and associate the virus status; ulceration status; factors of the TME (PD-L1 expression, E-cadherin expression and CD34 endothelial cells) and important immune cells in primary MCC and link these factors to disease-specific survival.

Importantly, we found that ulceration is an independent negative prognostic marker for patients with MCC. In melanoma, ulceration is a part of staging and is an established negative prognostic marker [22]; however, only few studies have looked at its role in MCC. Several studies have found no association [23,24,25,26], while Bob et al. found correlation between ulceration and poor MCC-specific survival [27]. Important limitations of many of these studies include a low number of ulcerated samples, unclear definition of ulceration or if analysis was performed on primary or metastatic tumors. In this study, ulceration was present in 29.5% (23/55) of primary tumors, with previous reports ranging between 6.7–40% [23,24,25,26,28]. Ulceration associated with absence of the virus and a high N:CD8, with the latter suggesting that ulceration may contribute to a tumor-supporting microenvironment by attracting neutrophils to the wound and surrounding tumor cells, in line with what has been previously shown in melanoma [16,29]. Neutrophils, inflammation and UV exposure can suppress the levels and functions of CD8 lymphocytes and induce inflammation and a local immune-suppressive microenvironment [30,31]. An alternative explanation may be that virus-negative tumors are larger and, therefore, more likely to be ulcerated; however, in our cohort, there was no significant difference in tumor size based on viral or ulceration status.

In our study, a virus-positive status estimated by qPCR associated with improved MCC-specific survival, confirming the results of several studies [32,33], although a virus-positive status estimated by IHC did not impact survival significantly (data not shown). In our cohort, 47% (43/90) of primary MCC samples were virus-positive in line with aggregate studies demonstrating 76% (453 of 595 MCCs) virus positivity, although ranges vary between 24% and 100% [32,34,35]. This variance is largely unexplained, as the hypothesis that this may be due to viral degradation in old FPPE patient samples has been rejected by digital transcriptome analysis of frozen virus-negative samples [36,37]. In support of our results, we used the same viral primers as previous published studies, and our bimodal approach of detecting the virus showed high concordance [35].

E-cadherin is an important adhesion molecule, and its loss is among the factors that are downregulated in epithelial-to-mesenchymal transition, allowing tumor cells to migrate [17,38]. In our sample, a reduced E-cadherin area fraction associated with virus-negative patients. This was unexpected, as virus-negative patients more often present with advanced disease, compared with virus-positive patients (66.7% vs. 48.3%) [32]. This is the first time E-cadherin expression has been linked to virus-negative status, and it may be rooted in the controversies regarding the cellular origin of MCC. Recent studies suggest that virus-positive MCC may originate from the epidermal keratinocyte, and virus-negative MCC may originate from the dermal fibroblast [39]. Based on these results, the difference in E-cadherin expression may be an intrinsic trait of each MCC host cell. An alternative explanation may be that the increased E-cadherin stain area fraction is an extrinsic, viral-mediated trait. Virus-mediated downregulation of E-cadherin has been reported for the Epstein-Barr virus in nasopharyngeal carcinoma and for the hepatitis C virus in hepatocellular carcinoma [40,41]. Future experiments with the knockdown of viral proteins may provide additional knowledge to this question.

The positive prognostic impact of CD8 lymphocytes and its association with PD-L1 is well-recognized [15,42,43]. The latter is well-known to occur through a CD8 lymphocyte-mediated induction of the interferon-γ pathway [44]. However, to the best of our knowledge, this is the first time that the N:CD8 ratio in the TME has been examined in MCC. In this current study, with 89 patients included in the analysis, a high N:CD8 ratio in the tumor was an independent prognostic marker of poor MCC-specific survival in both univariate and multivariate analysis. One recently published study examined its role in the peripheral blood of MCC patients, where a high N:CD8 ratio at baseline associated with a poor MCC-specific survival [45]. This may be due to the role of neutrophils in suppressing the antitumor effect of lymphocytes [30].

Our study had several important limitations, including its retrospective design. Ninety included patients in our analysis represent a large number in the scope of MCC research but is a relatively small sample size in statistical analysis. Formalin-fixed paraffin-embedded (FFPE) blocks were obtained from different pathology departments with different protocols from the time of tissue excision to final tissue preparation. We were therefore unable to control for the difference in fixation time, which could potentially affect the IHC. We used a digital image analysis that measures the immune stain area while manual assessments involve counting the number of stained cells, although comparative studies of these two evaluation methods show high concordance [46]. The strict legislation on the acquisition of patient journal materials meant that we could not obtain information on patient treatments. This may be a confounder when evaluating prognostic markers. Tumor size was not a prognostic marker in our cohort. This might be rooted in several factors, including the size and composition of our cohort, and may subsequently limit our findings. Due to the previous reported and accepted prognostic role of tumor size, we found it most correct adjusting for both lymph node involvement and tumor size in the multivariate analyses [1].

## 4. Materials and Methods

### 4.1. Patients and Samples

Patients diagnosed with MCC between 1 January 1996 to 31 December 2012 at Aarhus University Hospital and between 1 January 2007 to 31 December 2012 at Aalborg University Hospital, Vejle Hospital, Odense University Hospital, Herlev & Nordsjaelland Hospital, Bispebjerg Hospital and Rigshospitalet were included while searching the Aarhus Pathology Database and the Danish National Pathology Database using the SNOMED code M8247* for Merkel cell tumors. One-hundred and twenty-one (*n* = 121) patients matched the search criteria. After exclusion, ninety (*n* = 90) patients were included in the analyses (Figure S3). Clinical endpoints including the time of death and cause of death were obtained from the Danish Register of Causes of Death filed by a local doctor with knowledge of the patient’s admissions and disease history. Data on tumor size and pathology-confirmed regional lymph node involvement (fine needle aspiration and sentinel lymph node biopsy) were obtained from the Danish Pathology Database. This project was approved by the regional central Denmark Ethics Committee (Ethics code: 1-10-72-280-16)

### 4.2. Tumor Specimens

Formalin-fixed paraffin-embedded (FFPE) tissue blocks with primary MCC were evaluated at the Department of Pathology, Aarhus University Hospital. To confirm the diagnosis and presence of tumor tissues, 2-μm-thick sections were cut and stained with haematoxylin and eosin (HE) and evaluated by the departments senior pathologist (TS). Serial sections for further analysis with IHC and macro-dissections for DNA extraction were prepared.

### 4.3. DNA Extraction and Quantification

A 2-μm-thick section was cut and H&E-stained to mark a representative tumor-only area to guide the macro-dissection. Three sections (10-μm-thick) were cut and macro-dissected of the slide into a sterile tube. Between each patient sample, the microtome, gloves and knife were changed to avoid cross-contamination. DNA extraction was performed on the QIAsymphony SP (QIAGEN, Germany) following the manufacturer’s protocol. DNA purity and quantity were estimated on the Implen nanophotometer (Implen GmbH, Germany).

### 4.4. Real-time Taqman Polymerase Chain Reaction

Real-time quantitative PCR was performed on the Stratagene Mx3000P at the Department of Pathology, Aarhus University Hospital with previously tested Taqman viral primer sets (LT2, LT3, Set6 and Set7) with Onyx Quencher A (Sigma-Aldrich Company, Ltd, St. Louis, MO, USA) [35]. These primers are designed to amplify sequences within nucleotide position 196–1257 in the MCV genome. This region is known to be present in all variations of sequenced MCV-DNA from MCC. The housekeeping gene TATA-binding protein (TBP) was used as a reference (LGC Biosearch Technologies, United Kingdom; forward primer CACCACAGCTCTTCCACTCA; reverse primer GGGGAGGGATACAGTGGAGT; Probe AGACTCTCACAACTGCACCCTTGC). The testing was done with duplicates of each patient sample, negative controls (H_2_O, tonsillar tissue) and positive control with a Merkel cell virus-positive cell line (MKL-1, Sigma-Aldrich). qPCR was performed for 40 cycles at 95 °C for 3 s and 60 °C for 20 s.

### 4.5. Immunohistochemical Staining

**Figure 1:**
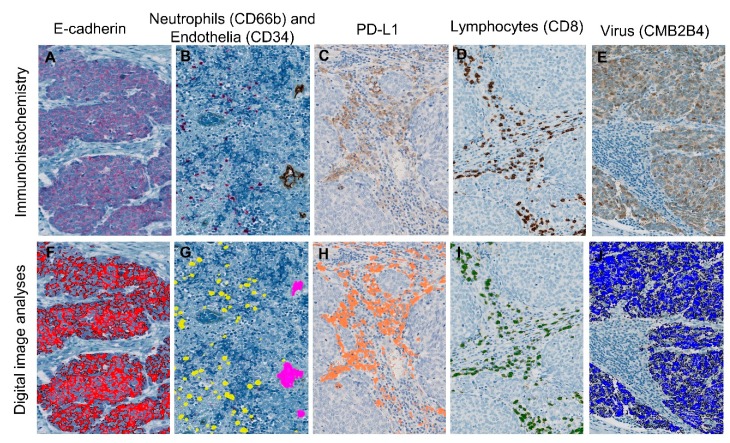
Sections stained with immunohistochemistry (IHC) (top row) analyzed with digital pathology (bottom row). Stained IHC sections of (**A**) E-cadherin, (**B**) CD66b neutrophils & CD34 endothelia, (**C**) PD-L1, (**D**) CD8 lymphocytes and (**E**) CMB2B4 at 20× magnification with comparable illustrations of digital image analysis (**F**–**J**). (**B**) CD34 endothelia (brown) and CD66b neutrophils (red) are stained on the same section. The digital image analysis software converts the IHC dye into a digital color that is used for the calculation of stain area fraction.

IHC was performed on the Ventana Benchmark XT-automated immunohistochemistry platform (Oro Valley, AZ, USA) and the Dako Autostainer Link48 (Santa Clara, CA, USA). From each FFPE, five consecutive sections (3-μm-thick) were cut and prepared for staining of CD8 lymphocytes (Dako, C8/144b, 1:200, OV dab); PD-L1 (Dako, 22C3, RTU, Dab); CD34 endothelia (Ventana, Oro Valley, AZ, USA, QBEnd/10, RTU, OV dab); CD66b neutrophils (BD Bioscience, Franklin Lakes, NJ, USA, G10F5, 1:200, UV red); E-cadherin (Ventana, Oro Valley, AZ, USA, 36, RTU, UV red) and CMB2B4 virus antigen (Santa Cruz, CA, USA, Poly, 1:100, OV dab) (Figure 1A–E). IHC was performed in large batches to reduce batch-to-batch variance between runs. Control tissue with internal negative and positive controls were used for all IHC staining. Control tissue for CMB2B4 virus antigen consisted of an MCV-positive patient sample estimated by qPCR and CMB2B4 staining, while tonsillar tissue was used for the remaining IHC stains.

### 4.6. Digital Pathology

Software from Visiopharm (Visiopharm A/S, Denmark) was used to attain a quantitative estimate of all analyzed factors. Image analysis protocols were developed by training the software to recognize specific colors of the stains used (Figure 1F–J). The results of image analyses of all sections were reviewed by the observer to exclude errors. A region of interest for the automatic evaluation of IHC stains was manually marked. The region of interest included tumor epithelium and adjacent intratumoral stroma. In this region, CD8, PD-L1, cd66b (both intra- and extravascular neutrophils) and CD34 (vascularization) were assessed, whereas E-cadherin and CMB2B4 (virus) were assessed only in the contained tumor epithelium. The IHC-stain area fraction per region of interest in percent was calculated regarding CD8 lymphocytes, PD-L1 and CD34 (vascularization), whereas the stain area fractions of virus-positive cells and E-cadherin were defined as the area of CMB2B-positive and E-cadherin-positive MCC cells, respectively, divided by the area of tumor epithelium. The tumor neutrophil-to-CD8 lymphocyte ratio (N:CD8 ratio) was estimated by the stain area fraction of cd66b divided by the stain area fraction of CD8 in the tumor.

### 4.7. Ulceration Status

Ulceration was defined as the full-thickness loss of the epidermis overlying MCC tissue in which epidermal loss was associated with a host reaction. The H&E-stained section was used for ulceration estimation, which was consensual based between SN and MLB, verified if in doubt by a senior pathologist (TS).

### 4.8. Viral Status

IHC: The Allred scoring system combines the intensity of staining (0–3) and proportion of cells stained (0-5), into a 0–8 points score. This method of semiquantative evaluation has previously been used to determine if a sample is considered positive for the MCV antigen, with a threshold set to 2 equating < 1% of cells with weak staining [32,47]. With this threshold in mind, the objective estimate in Visiopharm was set to analyze the stain area fraction of virus-positive cells with 1% as the cut-off.

qPCR: MCV is part of the skin flora and may therefore be present in tissue samples with virus-negative MCC [48]. To match the cut-off of immune staining, samples with less than 1% of cells containing viral DNA were categorized as ”PCR-negative” (equating a viral primer/TBP ratio < 0.01). In this study, virus status was based on the qPCR results.

### 4.9. Statistical Methods

The stain area faction of CD8 lymphocytes, PD-L1, neutrophils, CD34 endothelial cells, E-cadherin and virus antigen expression were log transformed, and the assumption of normal distribution assessed using the residuals. Correlations between the different markers were analyzed using linear regression and estimation of spearman correlation coefficients, and the differences in means between the groups were tested using a *t*-test. Data concerning the viral status (qPCR and immune staining) and ulcerated status was dichotomized and tested with a chi-square test. The study endpoint was disease-specific survival, defined as the time from the date of surgery to date of death from MCC. Statistical analysis of survival was performed using the Cox proportional hazards. Each variable was tested in multivariate analysis adjusted for two variables (tumor size and lymph node involvement) to retain sufficient statistical power with *n* > 10 events per adjusted factor. These variables are known prognostic markers in MCC, included in the 8th AJCC staging system [1]. Survival probabilities were illustrated using the Kaplan–Meier method. Level of significance of 0.05 was used for all analyses.

## 5. Conclusions

The results of this study show that patients with ulcerated primary tumors, absence of virus, scarce infiltration of CD8 lymphocytes and a high N:CD8 ratio have a significantly worse prognosis. In the clinical setting, we therefore suggest that these factors should be reported, as this may provide a more accurate prognosis and lead to better prognostic stratification for the patients in determination of the resection margin size and in the stratification of patients for adjuvant treatment based on the predicted risk of recurrence and death. Furthermore, estimation of ulceration status is easy, fast and does not require additional staining, while detection of virus, neutrophils and CD8 lymphocytes with IHC is reliable and easy to implement in the clinical labs.

## Figures and Tables

**Table 1:** This stain area fraction (in %) of immune cells and biomarkers in virus-positive and -negative Merkel cell carcinoma (MCC).

Mean Area Marker (%)	Virus-Positive MCC Mean Area Fraction of Marker (95% CI)	Virus-Negative MCC Mean Area Fraction of Marker (95% CI)	*p*-Value
Lymphocytes (CD8, intratumoral)	0.23 (0.06–0.89)	0.06 (0.02–0.19)	*p* = 0.11
PD-L1 (intratumoral)	59.28 × 10^−3^ (9.46 × 10^−3^–371.29 × 10^−3^)	4.36 × 10^−3^ (0.84 × 10^−3^–22.68 × 10^−3^)	*p* = 0.03
Neutrophils (CD66b, intratumoral)	0.19 × 10^−3^ (0.07 × 10^−3^–0.52 × 10^−3^)	0.89 × 10^−3^ (0.19 × 10^−3^–4.07 × 10^−3^)	*p* = 0.09
Neutrophil-to-lymphocyte ratio, (CD66b/CD8, intratumoral) *	0.81 × 10^−3^ (0.16 × 10^−3^–4.12 × 10^−3^)	15.93 × 10^−3^ (2.20 × 10^−3^–115.16 × 10^−3^)	*p* = 0.02
E-cadherin (intratumoral)	0.27 × 10^−3^ (0.04 × 10^−3^–2.04 × 10^−3^)	56.57 × 10^−3^ (6.44 × 10^−3^–497.02 × 10^−3^)	*p* = 0.0005
Endothelia (CD34, intratumoral)	3.74 (0.65–21.39)	4.40 (1.23–15.78)	*p* = 0.87

* No unit.

**Table 2:** Univariate analysis showing MCC-specific survival based on immune cells and biomarkers in the tumor microenvironment.

Characteristics	Number of Patients (*n*)	Univariate Analysis HR (95% CI)	*p*-Value
Presence of virus	90	0.47 (0.22–1.00)	*p* = 0.05
Presence of ulceration	78	2.49 (1.18–5.25)	*p* = 0.02
Lymphocytes (CD8, intratumoral)	90	0.70 (0.57– 0.87)	*p* = 0.001
Neutrophils (CD66b, intratumoral)	89	1.10 (0.91–1.34)	*p* = 0.32
Neutrophil-to-lymphocyte ratio (CD66b/CD8, intratumoral)	89	1.21 (1.06–1.37)	*p* = 0.004
Endothelia (CD34, intratumoral)	89	0.97 (0.82–1.15)	*p* = 0.74
E-cadherin (intratumoral)	89	0.98 (0.88–1.10)	*p* = 0.73
PD-L1 (intratumoral)	38	0.81 (0.59–1.12)	*p* = 0.21

**Table 3:** Multivariate analysis showing MCC-specific survival based on immune cells and biomarkers in the tumor microenvironment.

Characteristics	Number of Patients (*n*)	Multivariate Analysis HR (95% CI)	*p*-Value
Presence of virus	82	0.32 (0.13–0.78)	*p* = 0.01
Presence of ulceration	70	2.22 (0.99–4.98)	*p* = 0.05
Lymphocytes (CD8, intratumoral)	82	0.68 (0.54–0.85)	*p* = 0.001
Neutrophils (CD66b, intratumoral)	81	1.02 (0.82–1.26)	*p* = 0.87
Neutrophil-to-lymphocyte ratio (CD66b/CD8, intratumoral)	89	1.14 (1.00–1.31)	*p* = 0.04
Endothelia (CD34, intratumoral)	81	1.03 (0.86–1.23)	*p* = 0.77
E-cadherin (intratumoral)	81	0.98 (0.86–1.11)	*p* = 0.73
PD-L1 (intratumoral)	31	0.80 (0.53–1.20)	*p* = 0.29

## Supplementary Materials

The following are available online at https://www.mdpi.com/2072-6694/12/4/888/s1: Table S1: Characteristics between ulcerated and nonulcerated MCC, Figure S1: Images showing differences in staining and survival between ulcerated and nonulcerated MCC, Figure S2: Images showing differences in staining and survival between virus-positive and -negative MCC, Figure S3: Flowchart of included and excluded patients and samples.
