# Supplementary material for: Tumor Ulceration, Reduced Infiltration of CD8-Lymphocytes, High Neutrophil-to-CD8-Lymphocyte Ratio and Absence of MC Virus are Negative Prognostic Markers for Patients with Merkel Cell Carcinoma
Source: Cancers (Basel). 2020 Apr 6;12(4):888. doi: 10.3390/cancers12040888 (PMC7226107; doi:10.3390/cancers12040888)
Supplement: Supplementary file 1 [file cancers-12-00888-s001.pdf]

Supplementary Materials:

Tumor Ulceration, Reduced Infiltration of CD8-Lymphocytes, High Neutrophil-to-CD8-Lymphocyte Ratio and Absence of MC Virus Are Negative Prognostic Markers for Patients with Merkel Cell Carcinoma

Simon Naseri, Torben Steiniche, Jeanette Bæhr Georgsen, Rune Thomsen, Morten Ladekarl, Martin Heje, Tine Engberg Damsgaard and Marie Louise Bønnelykke-Behrndtz

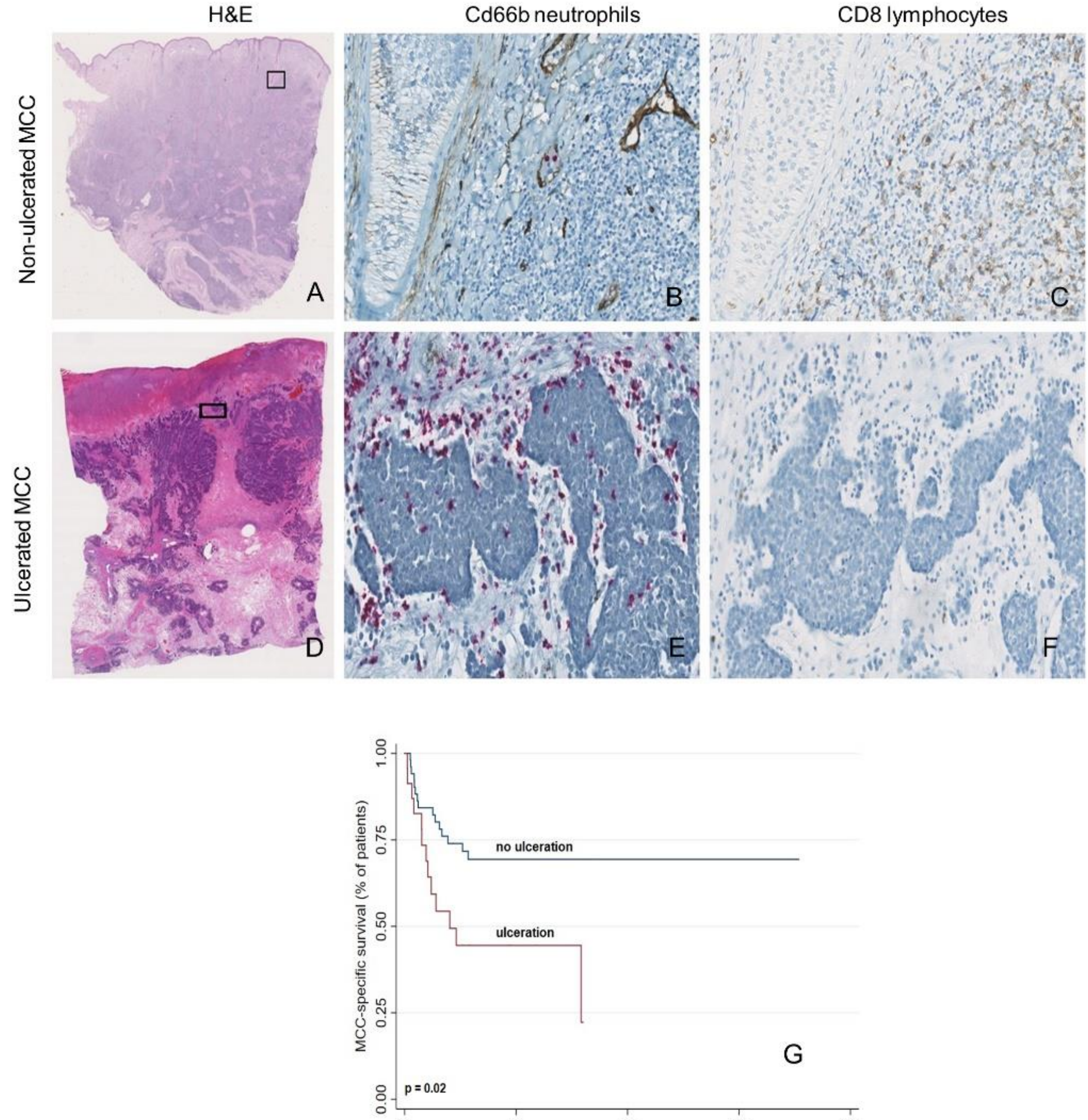

Figure S1. HE-stained section of a non-ulcerated MCC at 0.3x magnification (**A**). Same non-ulcerated MCC stained with CD66b neutrophils (**B**) and CD8 lymphocytes (**C**) at 20× magnification illustrating

absence of neutrophils and infiltration of CD8 lymphocytes. HE-stained section of an ulcerated MCC at 0.3x magnification (**D**). Same ulcerated MCC stained with CD66b neutrophils (**E**) and CD8 lymphocytes (**F**) at 20× magnification illustrating presence of neutrophils and virtual absence of CD8 lymphocytes. Kaplan-Meier curve showing MCC-specific survival between ulcerated and non-ulcerated MCC (**G**).

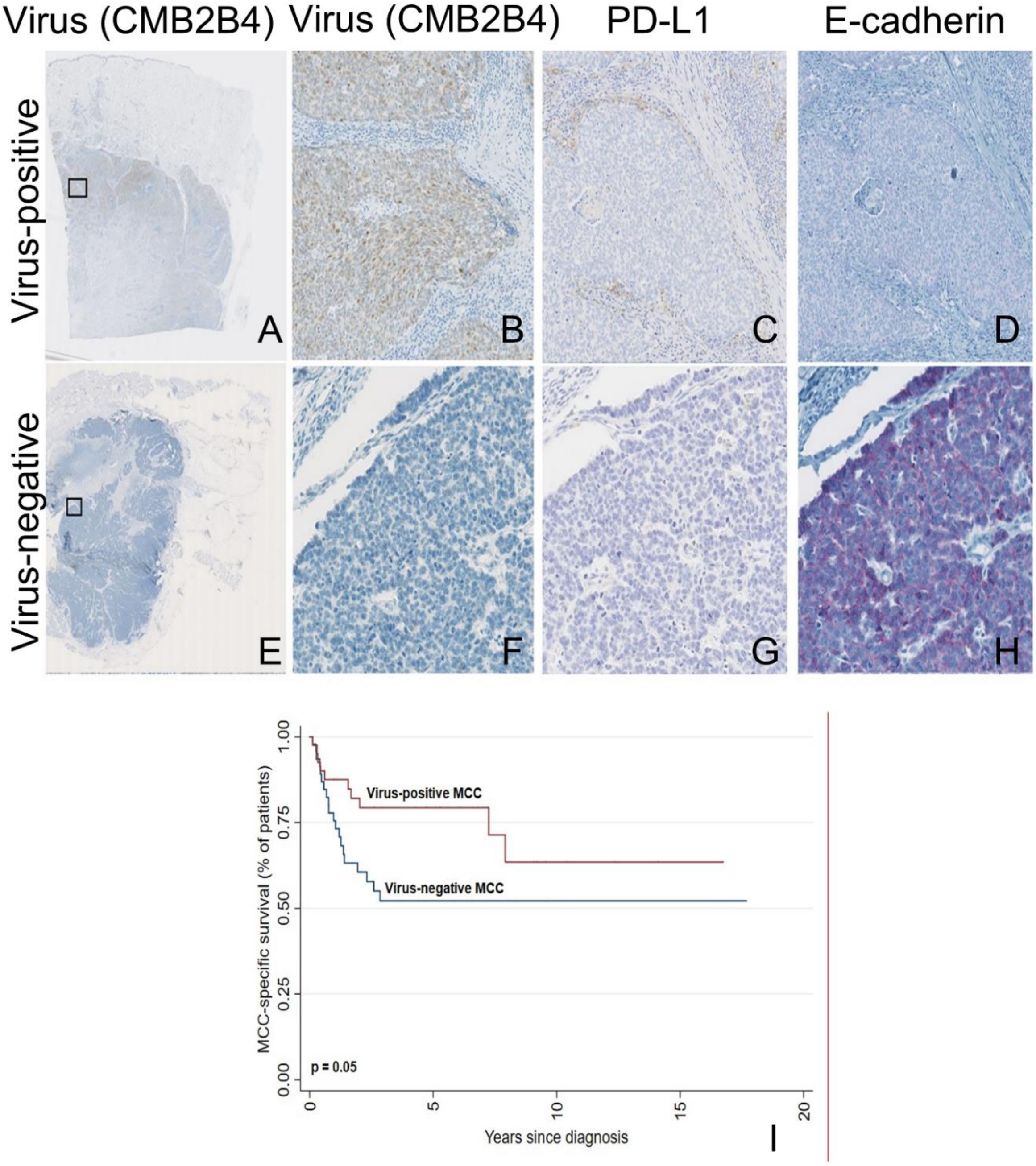

Figure S2. Virus-positive MCC stained with CMB2B4 at 0.3× magnification (**A**). Same virus-positive MCC slide at 20× magnification stained with CMB2B4 (**B**), PD-L1 (**C**) and E-cadherin (**D**) illustrating presence of virus, increased expression of PD-L1 and low expression of E-cadherin. Virus-negative MCC stained with CMB2B4 at 0.3× magnification (**E**). Same virus-negative MCC at 20× magnification stained with CMB2B4 (**F**), PD-L1 (**G**) and E-cadherin (**H**) illustrating absence of virus and PD-L1, but high expression of E-cadherin. Kaplan-Meier curve showing MCC-specific survival between virus-positive and virus-negative MCC (**I**)

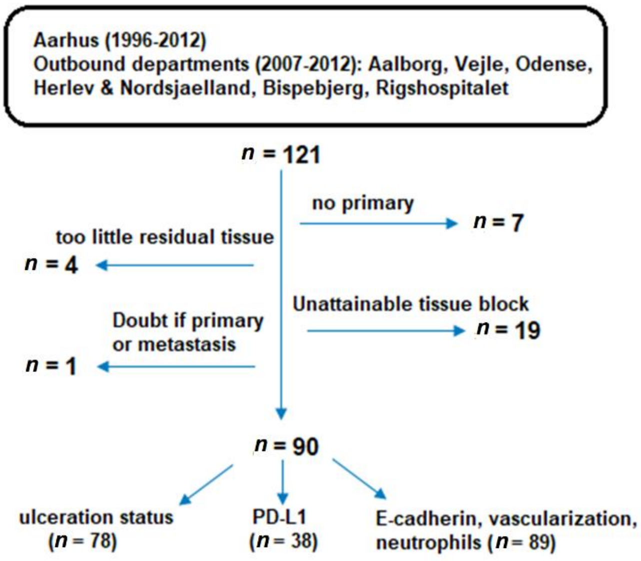

Figure S3. Flowchart illustrating selection of the final included and analyzed patient samples (*n* = 90) after excluding patients with no primary (*n* = 7), doubt if primary or metastatic MCC (*n* = 1), too little residual tissue (*n* = 4) or unattainable tissue blocks (*n* = 19). qPCR for virus status and immune histochemical staining (IHC) for CD8 lymphocytes and virus antigen (CMB2B4) was performed on all 90 samples. IHC staining for E-cadherin, endothelial cells and neutrophils were evaluated in 89 samples, as 1 sample ran out of material during the other analysis. Ulceration status could not be evaluated in 12 samples due to missing epidermis in the tumor sections. IHC staining for PD-L1 was performed on 38 randomly selected samples.

Table S1. Characteristics between ulcerated and non-ulcerated MCC.

Clinical Characteristics	Ulcerated (*n*=23)	Non-Ulcerated (*n*=55)	*P*-Value
	Sex		$p = 0.47$
Male	34.8% (8/23)	43.6% (24/55)	
Female	65.2% (15/23)	56.4% (31/55)	
Age at diagnosis (years), mean	81.3	76.6	$p = 0.07$
	Location		$p = 0.72$
Truncus	8.7 % (2/23)	9.1% (5/55)	
Upper/lower limb	47.8 % (11/23)	38.2% (21/55)	
Head and neck	43.5% (10/23)	52.7% (29/55)	
	Tumor size		$p = 0.56$
≤ 2 cm	55.6% (10/22)	52.8% (28/53)	
> 2 cm	44.4% (12/22)	47.2% (25/53)	
	Nodal Status		$p = 0.28$
Negative	63.6% (14/22)	76% (38/50)	
Positive	36.4% (8/22)	24% (12/50)	

© 2020 by the authors. Licensee MDPI, Basel, Switzerland. This article is an open access article distributed under the terms and conditions of the Creative Commons Attribution (CC BY) license (http://creativecommons.org/licenses/by/4.0/).
